# Editorial: Tailored respiratory muscle training for athletes, patients, and vulnerable groups

**DOI:** 10.3389/fspor.2025.1704447

**Published:** 2025-10-06

**Authors:** Felipe Contreras-Briceño, Fúlvia B. Manchado-Gobatto, Szczepan Wiecha

**Affiliations:** ^1^ Escuela de Kinesiología, Facultad de Salud, Universidad Santo Tomás, Arica, Chile; ^2^ Laboratory of Applied Sport Physiology, School of Applied Sciences, University of Campinas, Limeira, São Paulo, Brazil; ^3^ Hypoxia, Sport and Health Team - HSHT, School of Applied Sciences, University of Campinas, Limeira, São Paulo, Brazil; ^4^ Department of Physical Education and Health in Biala Podlaska, Faculty in Biala Podlaska, Jozef Pilsudski University of Physical Education, Warsaw, Poland; ^5^ Clinical Cardiology Centre, National Medical Institute of the Ministry of the Interior and Administration, Warsaw, Poland

**Keywords:** respiration, exercise, training, muscle, performance, therapy

**Editorial on the Research Topic**
Tailored respiratory muscle training for athletes, patients, and vulnerable groups

Research indicates that the maximum exercise capacity achieved in physical assessment tests is a leading indicator of population survival (1–4). To enhance physical capacity for both health professionals and sports science experts, it is essential to identify the biological systems that primarily limit exercise progression.

The goal is to design rehabilitation and physical conditioning programs that improve these “weaker” systems. In healthy individuals under normal conditions, the cessation of exercise is typically attributed to limitations in the cardiovascular system, particularly the inability to maintain an increase in cardiac output, as stroke volume tends to plateau at about 50% of maximum physical capacity (5–7).

However, in specific situations—such as extreme environments like high altitude or high humidity, clinical settings with ventilatory support or supplemental oxygen, and among populations like elite athletes, older adults, or patients with chronic diseases or cancer—other biological systems may play a crucial role (8, 9).

In this context, understanding the respiratory system is vital. Measuring respiratory mechanics, lung volumes through spirometry, and the strength and endurance of the respiratory muscles are important factors to consider when evaluating the respiratory system's contribution to physical performance in athletes and patients (10).

As a result, respiratory muscle training (RMT) has gained significant attention in health and sports sciences. Empirical evidence demonstrates that RMT can lead to improvements in clinical outcomes such as exercise capacity (including oxygen consumption and enhanced performance in tests like the six–minute walking test or shuttle walking test), reduced dyspnea and leg fatigue, and improved sports performance (11, 12).

Several studies have implemented different RMT protocols using a range of equipment and respiratory muscle tests to improve the program training prescription, all possessing high validity and reliability in accordance with international guidelines set by recognized organizations, such as the American Thoracic Society (ATS) and the European Respiratory Society (ERS) (13).

Acute interventions, such as warming up the inspiratory muscles, have also been suggested as strategies to enhance sports performance and improve the quality of subsequent physical exercise (14–16). Despite significant advances in the field of respiratory muscle training, the wide variety of methods and intervention protocols employed has posed challenges to reaching a consensus on the most effective approaches in this research and application area.

This research topic encompasses relevant studies on RMT in athletes and clinical populations, including ten submitted articles—five accepted for publication and five rejected. It underscores a significant interest in this area among health professionals and trainers who aim to optimize training by customizing RMT strategies. The focus is on identifying the most effective types, devices, and protocols to enhance these outcomes.

The contributions presented here primarily take the form of reviews, highlighting RMT's effectiveness on exercise performance in athletes and achieving noteworthy improvements in clinical populations. These contributions detail the devices used, the types of protocols implemented, and the reported values for the minimal clinically important difference (MID).

Despite the considerable variability in the evidence, there is a strong consensus on the utility of RMT within physical training programs. These findings convey a central message: regardless of the device used, RMT should be an integral component of all rehabilitation programs and sport contexts. While it is common to evaluate the effectiveness of an intervention based on outcomes relevant to the studied population, it would be misguided to confine the analysis of RMT to this framework.

The physiological response to physical effort is complex and involves integrating various “stressed” variables responding to physical stimulus. Therefore, the intricate interplay between biological systems during exertion requires deeper investigation. For example, improving respiratory mechanics through targeted training of respiratory muscles can enhance efficiency in effort expenditure, yielding benefits in other areas, such as the muscular system due to a delayed onset of metabolic reflexes, or cognitive aspects by mitigating exercise-induced hyperventilation and delaying the cerebral vasoconstriction associated with central fatigue.

As the advantages of RMT in training programs become evident, future research should concentrate on the integrated interactions of biological responses elicited by physiological stimuli and how to stimulate these responses without relying on costly instruments. We hope that this collection of research contributions will inspire new collaborations and encourage more researchers to innovate, aiming to make RMT more tailored, accessible, and recognized by health and sport professionals.

## References

[1] Oga T Nishimura K Tsukino M Sato S Hajiro T. Analysis of the factors related to mortality in chronic obstructive pulmonary disease: role of exercise capacity and health status. Am J Respir Crit Care Med. (2003) 167(4):544–9. 10.1164/rccm.200206-583OC 12446268

[2] Faselis C Doumas M Pittaras A Narayan P Myers J Tsimploulis A Exercise capacity and all-cause mortality in male veterans with hypertension aged ≥70 years. Hypertension. (2014) 64(1):30–5. 10.1161/HYPERTENSIONAHA.114.03510 24821944

[3] Kokkinos P Myers J Kokkinos JP Pittaras A Narayan P Manolis A Exercise capacity and mortality in black and white men. Circulation. (2008) 117(5):614–22. 10.1161/CIRCULATIONAHA.107.734764 18212278

[4] Kokkinos P Myers J Faselis C Panagiotakos DB Doumas M Pittaras A Exercise capacity and mortality in older men: a 20-year follow-up study. Circulation. (2010) 122(8):790–7. 10.1161/CIRCULATIONAHA.110.938852 20697029

[5] Fritzsche RG Switzer TW Hodgkinson BJ Coyle EF. Stroke volume decline during prolonged exercise is influenced by the increase in heart rate. J Appl Physiol. (1999) 86(3):799–805. 10.1152/jappl.1999.86.3.799 10066688

[6] Billat VL Petot H Landrain M Meilland R Koralsztein JP Mille-Hamard L. Cardiac output and performance during a marathon race in middle-aged recreational runners. TheScientificWorldJournal. (2012) 2012:810859. 10.1100/2012/810859 22645458 PMC3356747

[7] Murata M Adachi H Oshima S Kurabayashi M. Influence of stroke volume and exercise tolerance on peak oxygen pulse in patients with and without beta-adrenergic receptor blockers in patients with heart disease. J Cardiol. (2017) 69(1):176–81. 10.1016/j.jjcc.2016.02.017 27021429

[8] Bassett DR Jr Howley ET. Limiting factors for maximum oxygen uptake and determinants of endurance performance. Med Sci Sports Exerc. (2000) 32(1):70. 10.1097/00005768-200001000-00012 10647532

[9] Guazzi M Adams V Conraads V Halle M Mezzani A Vanhees L Clinical recommendations for cardiopulmonary exercise testing data assessment in specific patient populations. Circulation. (2012) 126(18):2261–74. 10.1161/CIR.0b013e31826fb946 22952317 PMC4777325

[10] Leahy MG Kipp S Sheel AW. The respiratory physiology of exercise: age and sex considerations. Current Opinion in Physiology. (2023) 33:100652. 10.1016/j.cophys.2023.100652

[11] Espinosa-Ramírez M Riquelme S Araya F Rodríguez G Figueroa-Martínez F Gabrielli L Effectiveness of respiratory muscles training by voluntary isocapnic hyperpnea versus inspiratory threshold loading on intercostales and vastus lateralis muscles deoxygenation induced by exercise in physically active adults. Biology (Basel). (2023) 12(2):219. 10.3390/biology12020219 36829497 PMC9953077

[12] Kowalski T Kasiak PS Rebis K Klusiewicz A Granda D Wiecha S. Respiratory muscle training induces additional stress and training load in well-trained triathletes-randomized controlled trial. Front Physiol. (2023) 14:1264265. 10.3389/fphys.2023.1264265 37841319 PMC10576561

[13] American Thoracic Society/European Respiratory Society. ATS/ERS statement on respiratory muscle testing. Am J Respir Crit Care Med. (2002) 166(4):518–624. 10.1164/rccm.166.4.518 12186831

[14] Marostegan AB Gobatto CA Rasteiro FM Hartz CS Moreno MA Manchado-Gobatto FB. Effects of different inspiratory muscle warm-up loads on mechanical, physiological and muscle oxygenation responses during high-intensity running and recovery. Sci Rep. (2022) 12(1):11223. 10.1038/s41598-022-14616-w 35780133 PMC9250525

[15] Cirino C Marostegan AB Hartz CS Moreno MA Gobatto CA Manchado-Gobatto FB. Effects of inspiratory muscle warm-up on physical exercise: a systematic review. Biology (Basel). (2023) 12(2):333. 10.3390/biology12020333 36829608 PMC9953131

[16] Soares de Araujo L Marostegan AB Menezes Scariot PP Bordon Orsi J Cirino C Papoti M Inspiratory muscles pre-activation in young swimmers submitted to a tethered swimming test: effects on mechanical, physiological, and skin temperature parameters. Sci Rep. (2024) 14(1):5975. 10.1038/s41598-024-52312-z 38472356 PMC10933374

